# Development of Thermal Principles for the Automation of the Thermographic Monitoring of Cultural Heritage

**DOI:** 10.3390/s20123392

**Published:** 2020-06-16

**Authors:** Iván Garrido, Susana Lagüela, Stefano Sfarra, Pedro Arias

**Affiliations:** 1 Applied Geotechnologies Research Group, Centro de Investigación en Tecnoloxías, Enerxía e Procesos Industriais (CINTECX), Universidade de Vigo, Campus Universitario de Vigo, As Lagoas, Marcosende, 36310 Vigo, Spain; ivgarrido@uvigo.es (I.G.); parias@uvigo.es (P.A.); 2 Cartographic and Land Engineering Department, Higher Polytechnic School of Ávila, University of Salamanca, Hornos Caleros 50, 05003 Ávila, Spain; 3 Department of Industrial and Information Engineering and Economics, University of L’Aquila, Piazzale E. Pontieri 1, Monteluco di Roio, I-67100 L’Aquila, Italy; stefano.sfarra@univaq.it

**Keywords:** InfraRed Thermography, thermal principles, cultural heritage, preservation, monitoring, mosaic, internal water, automation

## Abstract

The continuous deterioration of elements, with high patrimonial value over time, can only be mitigated or annulled through the application of techniques that facilitate the preventative detection of the possible agents of deterioration. InfraRed Thermography (IRT) is one of the most used techniques for this task. However, there are few IRT methodologies, which can automatically monitor the cultural heritage field, and are vitally important in eliminating the subjectivity in interpreting and accelerating the analysis process. In this work, a study is performed on a *tessellatum* layer of a mosaic to automatically: (i) Detect the first appearance of the thermal footprint of internal water, (ii) delimit the contours of the thermal footprint of internal water from its first appearance, and (iii) classify between harmful and non-harmful internal water. The study is based on the analysis of the temperature distribution of each thermal image. Five thermal images sequences are acquired during the simulation of different real situations, obtaining a set of promising results for the optimization of the thermographic inspection process, while discussing the following recommended steps to be taken in the study for future researches.

## 1. Introduction

The conservation of cultural heritage is an increasingly important field for all societies in the 21st century, given that heritage structures are key features in the history of humanity. Heritage buildings are evidence of the evolution of the human being, and each heritage element provides cultural, spiritual and aesthetic satisfaction and economic benefits to the region in which it is located, and consequently to its inhabitants [1]. Therefore, it is vitally important to address the continuous deterioration of heritage structures due to the appearance of different defects caused by ageing, unpredicted events, environmental conditions and incorrect previous restoration treatments [2]. To this end, cultural heritage monitoring is an appropriate solution, consisting of a process of evaluation and tracking the integrity of the heritage structure under study over time. Then, the advantage of using cultural heritage monitoring is that it makes possible to detect and characterize the nature of possible defects in the structure in their initial stages of growth [3]. Consequently, cultural heritage monitoring allows to apply the maintenance tasks required to slow down and even reduce the deterioration.

Different types of sensors can be used to analyse the integrity of a heritage structure for cultural heritage monitoring applications. The group of sensors that is currently emerging the most are those belonging to tools classified as Non-Destructive Techniques (NDTs) [4]. NDTs differ from destructive techniques by not intruding or affecting the integrity of the structure under study [5]. Therefore, NDTs allow an analysis with a high level of detail, such as the monitoring process, in any type of material, including the most delicate heritage elements such as paintings, sculptures and mosaics, among others.

One of the most profitable NDT for the non-destructive inspection of heritage structures is InfraRed Thermography (IRT) [6]. This tool gets the temperature of the surface of any material, after the conversion of the radiation measured and emitted by the body under study. Specifically, the radiation measured for temperature determination corresponds to one of the bands that divide the infrared spectrum (mostly Medium-Wave and Long-Wave InfraRed, MWIR and LWIR respectively), and it is received by sensors that are sensitive to this band, known as thermal sensors. The optimal wavelength to obtain temperature (the infrared spectrum is between 0.4 @ 1000 µm [7]) depends on the range of temperatures of the surface of the material. For instance, if the temperature of the material surface is in ambient conditions, the appropriate thermal sensors are those sensitive to the LWIR band (7 @ 14 µm) and if the temperature of the surface is greater than 100 or 150 °C, the thermal sensors that cover the MWIR band (3 @ 5 µm) are the recommended option [8].

Within the cultural heritage field, which elements are normally under ambient conditions, thermal sensors used are sensitive to the LWIR band and usually installed inside a camera by means of a plane array (thermographic camera). In addition to the NDT advantages, IRT has the following strong points for cultural heritage monitoring [7,9]:

- Remote and real time operation.
- High accuracy in the measured temperature values and high-speed scanning of the thermographic camera.
- Easily interpretable results in two-dimensional (2D) (thermal images).

A clear evidence of the usefulness of IRT in cultural heritage is the large number of published IRT works related to the conservation of different heritage structures. Among the most recent IRT review papers, Garrido et al. [7] and Moropoulou et al. [10] present several IRT applications to infrastructure inspections, among them heritage sites, and demonstrate the importance of IRT application at all stages of a conservation project, from diagnosis to evaluation of conservation interventions, for ordinary or large-scale conservation projects, respectively. In addition, Mercuri et al. [11] and Ibarra-Castanedo et al. [12] review the recent applications of a particular type of inspection, named Pulsed InfraRed Thermography, on different types of cultural heritage artefacts, and explain the theory of IRT in the context of cultural heritage studies showing also some case studies, respectively.

Regarding IRT papers of original research, Georgescu et al. [13] monitored with IRT the interior of a church to evaluate improvements made after restoration works in search of moisture areas to remove. Sfarra et al. [14] also analysed walls belonging to two aediculae, detecting different cracks with IRT, while Kilic [15] and Bisegna et al. [16] examined a historic Ottoman building and ancient façades, respectively, using the IRT technique. In [15], the presence of concrete deterioration, water seepage, cover delamination and significant cracks were detected and, in [16], the most relevant damages produced in the structures by an earthquake are revealed. Moreover, Solla et al. [17] detected moisture in a masonry arch bridge through Ground-Penetrating Radar (GPR), photogrammetry and IRT, and Garrido et al. [18] automatically delimited and geometrically characterized different moisture areas from three ancient walls. As for paintings, Cadelano et al. [19] assessed the decomposition of fresco mural painting inside a medieval chapel through the detection of the presence of water on the decorated surfaces and inside the walls, while Sfarra et al. [20] studied the state of a mural painting with IRT in combination with other NDTs and micro-destructive analytical techniques, identifying a sandwich structure having the interstitial void full of moisture.

Moisture stands as one of the main defects analyzed within the cultural heritage field, established in previous IRT work. The analysis of moisture using IRT is linked to the changes of the thermal properties provoked by the water between the defect and the unaltered surrounding of the heritage structure. This change in the thermal properties can cause a change of temperature between both volumes that can be detected by a thermographic camera [21]. The severity of this defect comes from the fact that it can cause several deterioration phenomena in the structure affected if maintenance tasks are not applied during its initial growth stage, such as mould growth, efflorescence/salt crystallization, cracking and detachment [22]. In the case of a mosaic, the presence of moisture, if severe, provokes the detachment of its components with respect to its underlying support due to the combination of chemical infiltration of the plaster with salts dissolved in the moisture. The reason for emphasizing mosaics as heritage structures is because of the long history and tradition of human beings in the construction of this type of elements, estimating that the first mosaics are 4000 years old or more [23]. Then, the conservation of different mosaics over time is of fundamental importance, attracting the interest of the scientific community including the IRT researchers. Proof of this are the several recently published IRT works, which detect defects, including moisture or characterize the state of degradation and/or the provenance of the superficial components from different types of mosaics [22,23,24,25,26,27,28,29,30,31].

Nevertheless, all the IRT papers that analyse mosaics or other types of heritage element, have a common factor; none propose an automatic inspection except [22,31]. What is more, with respect to automatic monitoring, only Garrido et al. [31] presented the first steps. [31] proposes the automatic detection and delimitation of different thermal footprints of internal water from a thermal image sequence, which was artificially generated and located within the *tessellatum* mosaic used in [23]. The advantages of automating the monitoring process include the fact that automation avoids the subjectivity of the interpretation of the data acquired by an operator and accelerates the analysis process, where the reduction of the consumption time is essential in monitoring applications.

Therefore, a study is performed in this paper, using the same sample of [31] and focusing on the following objectives:

1. The automatic detection of the moment of the first appearance of the thermal footprint of the internal water located within the mosaic from a thermal image sequence, being the time essential in the case of applying maintenance tasks.
2. The improvement of the process of the automatic delimitation of the contour of the thermal footprint of the internal water regarding the process proposed in [31], without only delimiting at the instant when the thermal footprint of the internal water is most representative, but also in the rest of the test due to the importance of knowing at all times the extension affected by moisture in the heritage structure.
3. The automatic classification of the two types of internal water studied in [31]. One is classified as internal water that does not damage the mosaic, i.e., circulation of water through internal pipes that is artificially generated by the recirculation of water thanks to a water pump. The other is classified as harmful, i.e., moisture propagation that is artificially generated by injecting water into an internal sponge. This type of classification is critical to differentiate false and non-false alarms to the operator who is reviewing the automatic monitoring.

To that purpose, this study starts from the basis of the method proposed in [31], focusing on the temperature distribution of each thermal image of the monitoring/sequence used as input data. Before the description of the analysis performed in the study (Section 3), the sample under study is described in the Section 2 in more detail, together with the experimental configuration and the monitoring performed. The thermal image sequence of each test simulates a different real situation that can be found in the sample regarding the appearance of one of the types of internal water under study (Section 2). This study ends by representing the results obtained from the analysis performed together with the corresponding discussion (Section 4) and closing by commenting on the conclusions (Section 5).

## 2. Materials and Methods

The sample of this work is a mosaic manufactured with different types of artificial defects, which are placed at different depths and positions. The materials that integrate the mosaic together with the inserted defects and the phases of construction of the sample are described with high level of detail in reference [23]. Then, this paper summarizes the information, highlighting the most interesting aspects according to the objectives of this work.

**Figure 1:**
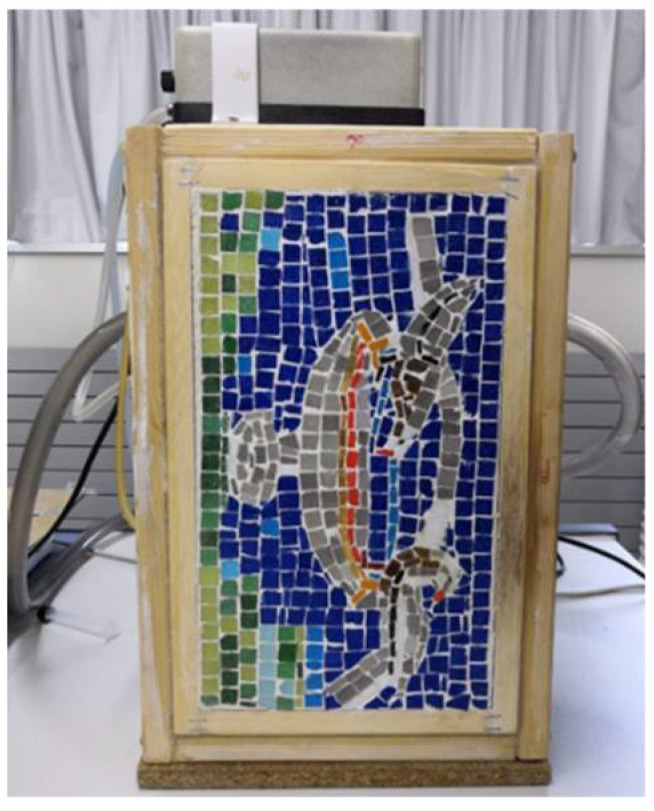
Front surface of the sample to be tested.

Figure 1 shows the front surface of the mosaic. As can be appreciated, the sample was executed in a wooden frame.

The first supporting sub-mosaic layer is natural mortar with the insertion of clay-brick pieces which is located in the layer at the lower side of the wooden frame (i.e., on the opposite side of the front surface of Figure 1). As for the intermediate layer, four artefacts are placed and covered by slurry, being only two of them analysed in this work. The first artefact under study is a transparent silicone duct passing through the sample which allows the continuous flow of water recirculated from a small tank placed at the bottom back of the mosaic and thanks to a water pump (hereinafter, the artefact is referred to as *water_pipe*). The water pump is placed just inside the tank, recirculating water with a constant flow rate of 420 L/h. The second artefact is a plastic sponge in contact with a silicone duct passing through only one lateral side of the wooden frame, inserting a known amount of water in the duct using a graduated syringe. The inserted water reaches the sponge with a flow rate equal to 18 L/h (hereinafter, the artefact is referred to as *water_sponge*).

These two artefacts are of interest and used in this paper since the first simulates the flow of circulation of water through internal pipes, and the second allows the study of water diffusion inside the mosaic, that is, the moisture propagation effect. Both simulations are performed under controlled thermo-physical conditions. In other words, *water_pipe* makes possible to simulate internal water that is not harmful to the heritage element, and *water_sponge* allows to simulate the propagation of harmful internal water.

**Figure 2:**
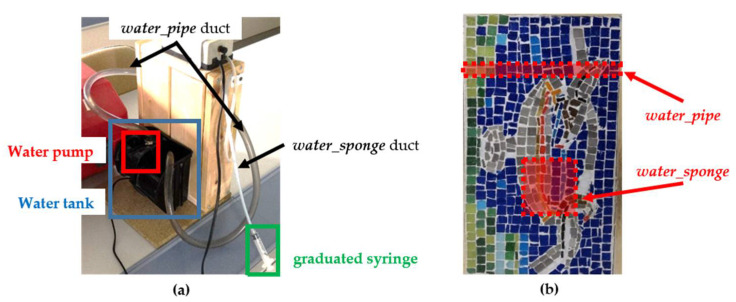
(**a**) Water tank, water pump, graduated syringe, *water_pipe* and *water_sponge* ducts positions; (**b**) *water_pipe* and *water_sponge* locations from the front surface of the mosaic.

**Figure 3:**
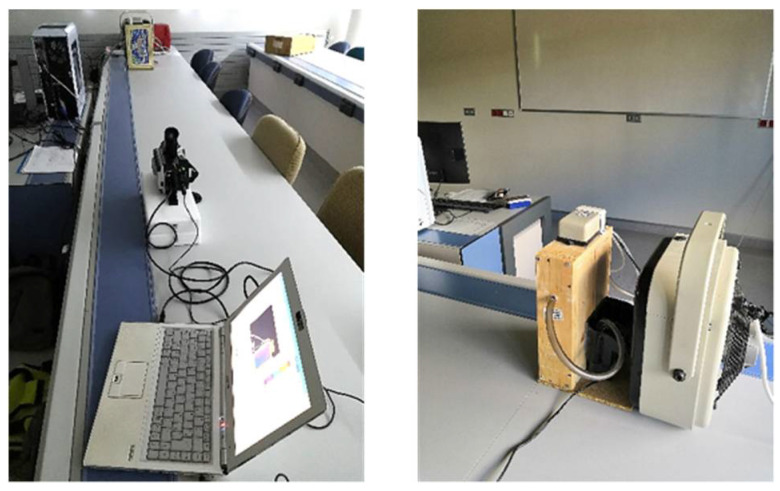
Experimental setup: relative positions of the sample, the stove and the thermographic camera, in transmission mode.

In the following two layers, two additional artefacts are included and also covered by slurry, being the surface finish of the mosaic a *tessellatum* layer composed by synthetic tesserae, i.e., different tiles made of a molten glass type, which is adhered to the slurry. The tiles used have different colours representing a reproduction of the mosaic of the doves site in Villa Adriana in Rome (Italy). Regarding the mosaic dimensions, the width and height are 0.16 m and 0.28 m, respectively. The width and height are in coherent with the position and orientation of the mosaic in Figure 1. For more information on the parameters related to the location and depth of each artefact, Figures 2 and 3 of reference [23] show the corresponding values.

To finish with the description of the sample, Figure 2a shows where the water tank and water pump are located, as well as the *water_pipe* and *water_sponge* ducts and the graduated syringe. In addition, Figure 2b indicates where *water_pipe* and *water_sponge* are positioned with respect to the front surface of the mosaic.

The experimental setup consists on the thermal excitation of the sample with a stove and the recording of the thermal response with a thermographic camera. The thermographic method used is “transmission mode” as shown in Figure 3. The stove used is 3.4 kW, and is placed at the rear of the mosaic, at 30 cm distance to uniformly heat the rear side. The thermographic camera is located 2.4 m from the front surface of the mosaic (the mosaic with the same orientation regarding Figure 1), and it is centred on the centroid of the sample to cover the entire front surface of the mosaic. The thermographic camera specifications are shown in Table 1.

Given the stove is close to the mosaic, this thermal source heats the air and not directly the surface, not being as thermally aggressive as other artificial external thermal sources, such as flash lamps. In addition, the stove is installed on the back side of the mosaic where a layer of wood is present. The wood layer has a low thermal conductivity and homogenizes the heat transfer on the x-y plane. In a real situation, the wood layer would be replaced by some construction material with similar thermal conductivity value. As for the interior of the mosaic, it also has a low thermal conductivity, where in a real case study there would also be insulation construction materials inside. In this way, any damage due to the heat generated is avoided, even on the precious face under study (i.e., the *tessellatum* layer), and it is not necessary to limit time intervals or temperature if this experimental configuration is used.

Moreover, five different tests are performed under laboratory conditions, all with a thermal image acquisition interval equal to 5 s. This acquisition interval has been selected as a balance between ensuring the measurement of all the temperature steps that can arise in the sample and the minimum amount necessary of thermal images in each test. Table 2 describes in detail the five tests performed_._

In the tests represented in Table 2, a heating period and a continuous cooling period are applied by the stove. In other words, by means of the stove a transitory heating with a continuous transitory cooling of a typical unstable day is simulated, considering the front surface of the mosaic located in a closed environment where ambient parameters are under control (such as inside a museum) and the rear surface in contact with the external environment. In this way, it is possible to prepare the data for the study proposed towards the automatic:

1. Detection of the first appearance of the thermal footprint of internal water in the sample (acting Test_5 as a comparison, since no internal water is generated).
2. Delimitation of the contour of the thermal footprint of the internal water detected for every image acquired, from the moment of the detection of its appearance, whether the internal water appears during a transitory cooling just after a transitory heating (Test_1 and Test_2), or if it appears during a transitory heating (Test_3 and Test_4).
3. Classification of the thermal footprint of the internal water detected and delimited between harmful (Test_2, Test_4) and non-harmful (Test_1 and Test_3) for the sample.

## 3. Data Analysis

The study is divided in three consecutive different steps, in accordance with each of the three objectives. These stages are detailed in the following subsections.

### 3.1. Step 1: Automatic Detection of the First Appearance of the Thermal Footprint of Internal Water

According to reference [31], it is assumed that the different thermal behaviour between a region with moisture, thermally affecting the surface of the material under study, and the rest of the unaltered surrounding of the material results in a pseudo-bimodal temperature distribution. That difference in thermal behaviour is due to the distinct values of the thermophysical properties that characterize each volume, i.e., the moisture region and the unaltered surrounding of the material under study [32]. This means that a defect will have its own Gaussian temperature distribution, which will appear in the histogram of the thermal image if it affects thermally the surface of the object analysed, i.e., there is a thermal footprint of the defect on that surface. This hypothesis was also demonstrated in other recent IRT papers besides reference [31], which focused on different defects, such as in the analysis of the thermal behaviour of moisture [22] and thermal bridges [33,34]. In addition, a small number of pixels will typically belong to the thermal footprint of a defect with respect to the total number of pixels of the thermal image, in the case of cultural heritage inspections. This leads to the maximum peak of the Gaussian temperature distribution of the thermal footprint of the defect is lower than the maximum peak of the continuous Gaussian temperature distribution belonging to the unaltered surrounding, i.e., the pseudo-bimodal temperature distribution. It should be noted that the Gaussian bell is the typical temperature distribution for thermal image histograms of defect-free materials. See reference [22] for more information and demonstrations.

Thus, in the Step 1 of the study it is deduced that at the moment when internal water is incorporated, the histograms of the thermal images will gradually evolve from a Gaussian temperature distribution to a pseudo-bimodal temperature distribution, regardless of whether the internal water damages the structure or not and whether it cools or heats the unaltered surrounding. In this case, the internal water cools the unaltered surrounding in all the tests according to the values of the ambient temperature, which will be similar to the mosaic temperature, and of the water temperature shown in Table 2. Then, the difference in the shape among the histograms of the thermal images over time is analysed in order to automatically detect the first appearance of the thermal footprint of internal water. Specifically, the slopes in the evolution of the sum of the absolute values of skew and kurtosis of each thermal image in a sequence are studied.

**Figure 4:**
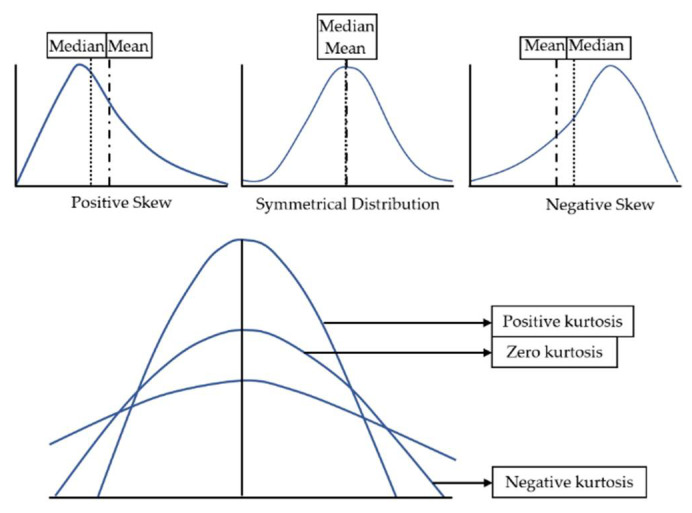
Definition of the possible types of skew and kurtosis in a dataset distribution. Graphical explanation.

The skew parameter is basically the degree of distortion of the distribution of a dataset (in this case, the pixel values of a thermal image) with regard to a symmetrical distribution, i.e., the farness or closeness to the zero skew-value, which is the corresponding value for a Gaussian bell. The kurtosis parameter consists of the analysis of the extreme values in a dataset distribution (in this case, again, the pixel values of a thermal image), i.e., between its tails relative to a Gaussian distribution [35]. Figure 4 shows graphically the explanation of this paragraph, and Equations (1) and (2) represent the formulas for calculating the skew and kurtosis parameters, respectively.

(1) $$\mathrm{Skew}=\frac{\sum_{i=1}^{N}{\left(Y_{i}-\bar{Y}\right)}^{3}}{N\times s^{3}}$$

(2) $$\mathrm{Kurtosis}=\frac{\sum_{i=1}^{N}{\left(Y_{i}-\bar{Y}\right)}^{4}}{N\times s^{4}}-3$$

where $Y_{i}$ is each data “*i*” of the dataset distribution (in this case, each pixel value of the thermal image), $\bar{Y}$ stands for the mean value of the dataset distribution (in this case, the mean value of the thermal image), *N* represents the total number of data of the dataset distribution (in this case, the thermal image resolution) and s is the standard deviation value of the dataset distribution (in this case, the standard deviation value of the thermal image).

To obtain automatically the slopes in the evolution of the sum of the absolute values of skew and kurtosis from a thermal image sequence, two processes are previously applied automatically in this Step 1. The first pre-processing is a noise filtering applied to each thermal image, using the technique known as bilateral filtering. This nonlinear function allows to remove most noise from an image, while preserving the edges of possible objects [36], i.e., the contour of the thermal footprint of the internal water. By doing so, the continuous increases and decreases of the evolution of the sum of skew and kurtosis due to the noise is mitigated, even though the noise is not eliminated in its entirety. Equation (3) mathematically represents the bilateral filtering [34],

(3) $$\mathrm{dst}(x,y)=\frac{1}{\sum_{x_{i},y_{i}}f_{r}\left(src\left(x_{i},y_{i}\right)-src\left(x,y\right)\right)\times g_{s}\left(x_{i}-x,y_{i}-y\right)\times \sum_{x_{i},y_{i}}\left(src\left(x,y\right)\times f_{r}(src\left(x_{i},y_{i}\right)-src\left(x,y\right)\right)\times g_{s}\left(x_{i}-x,y_{i}-y\right))}$$

where *x*, *y* are the coordinates of the current pixel of the thermal image to be filtered, *x_i_*, *y_i_* are the coordinates of the pixel neighbourhood that is used during filtering and defined by argument *d*, *src* is the input thermal image, *dst* is the thermal image after the bilateral filtering application, *fr* is a Gaussian filter depending on the intensity space and *g_s_* is a Gaussian filter depending on the coordinate space. The value of the argument *d*, and the sigma values used in *fr* and *g_s_* are the same as the values used in [34], given the good result offered (see Table 2 in [34]).

The second pre-processing is the Savitzky-Golay (SG) smoothing filter application, which is applied to the evolution of the sum of skew and kurtosis with the aim at allowing the computation of its slopes. This filter is based on the least-squares method, which main advantage is that it tends to preserve characteristics of the initial distribution such as relative maximum and minimum values while softening the intermediate values [37]. Equation (4) mathematically represents the generic form of this filter:

(4) $$y^{i}\left(n\right)=i!\times \overset{M}{\underset{m=-M}{\sum }}C_{i}\left(-m\right)\times x\left(n-m\right)\;,\;\;i=d$$

where $y^{i}\left(n\right)$ is the output of a *n* data of the dataset (in this case, one of the sum of skew and kurtosis) in the *i*-derivative, ! is the factorial symbol, *d* is the degree of the polynomial fit (in this case, it is calculated by the sum of the relative maximums and minimums obtained after applying the *find_peaks* function [38] to the evolution of the sum of skew and kurtosis), *M* is a natural number pointing symmetrical to each side of x_0_ and calculated as follows: *M* = (*N* − 1)/2, *N* is the length of the dataset (in this case, the total number of sums of the evolution), *C_i_* is the matrix of coefficients in the *i*-derivative that is optimally determined so that the corresponding polynomial curve best fits the dataset *x* (in this case, *x* is the evolution of the sum of skew and kurtosis).

Then, the automatic computation of the slopes is performed by: (i) The automatic search for the relative maximum and minimum values in the resulting evolution of the sum of skew and kurtosis after the SG smoothing filter application by the *find_peaks* function [38], (ii) the automatic calculation of the numerical value of the slope between each two consecutive extremes obtained in (i) ($∆y_{i}/∆x_{i}$, being $∆y_{i}$ and $∆x_{i}$ the difference between the values of two consecutive extremes and between the positions of those extremes, respectively), and (iii) the automatic calculation of the percentage value of the slope between each two consecutive extremes ($\frac{∆y_{i}/∆x_{i}}{\sum^{}\left|∆y\right|/\left|∆x\right|}\times 100$, being $∆y_{i}/∆x_{i}$ the numerical value of the slope *i* obtained in (ii), and ∑|∆y|/|∆x| the sum of all the numerical values of the slopes of the evolution obtained in (ii) in absolute terms).

**Figure 5:**
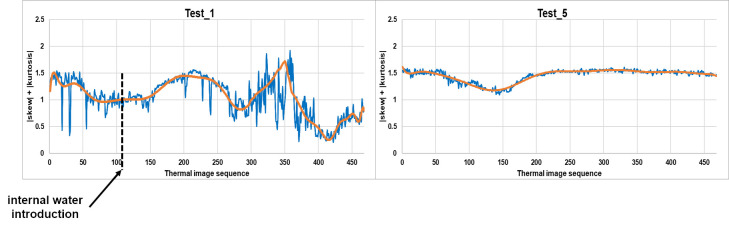
Evolution of the skew and kurtosis summation in Test_1 and Test_5. Before (blue line) and after (orange line) SG smoothing filter application.

**Figure 6:**
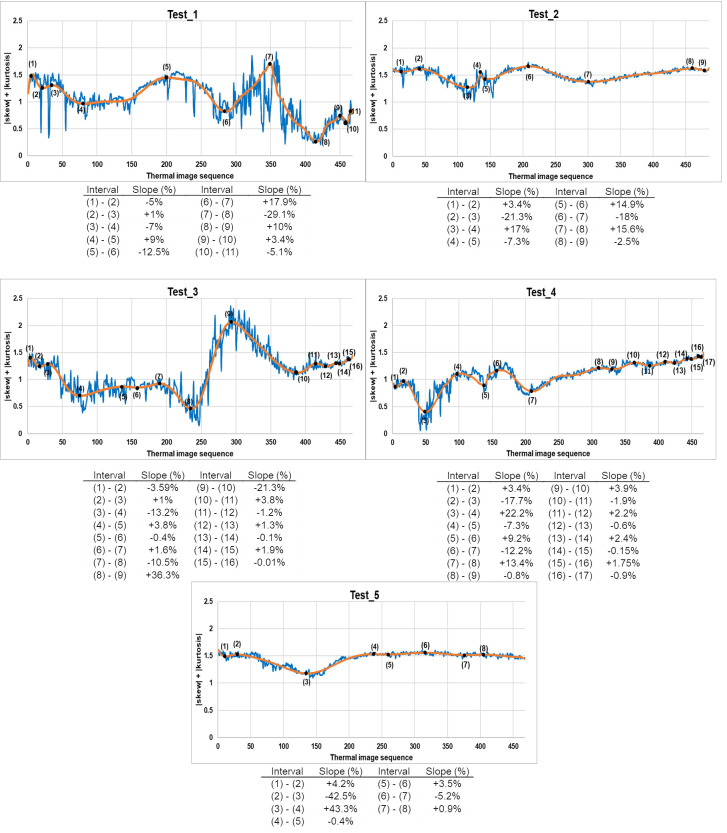
Relative maximums and minimums obtained (numbers indicated in the graph) and slope calculation (table below the graph) following the evolution of each test performed after SG smoothing filter application (orange line). Blue lines indicate the evolutions of the tests performed before SG smoothing filter application.

As an example, Figure 5 shows the corresponding evolutions of the skew and kurtosis summation in Test_1 (introducing internal water, at the instant corresponding to the thermal image 120 indicated by means of a discontinuous vertical line) and Test_5 (without internal water) before and after the SG smoothing filter application. Moreover, Figure 6 shows the resulting relative maximums/minimums and slopes, in %, of each test used as input data in this work.

**Figure 7:**
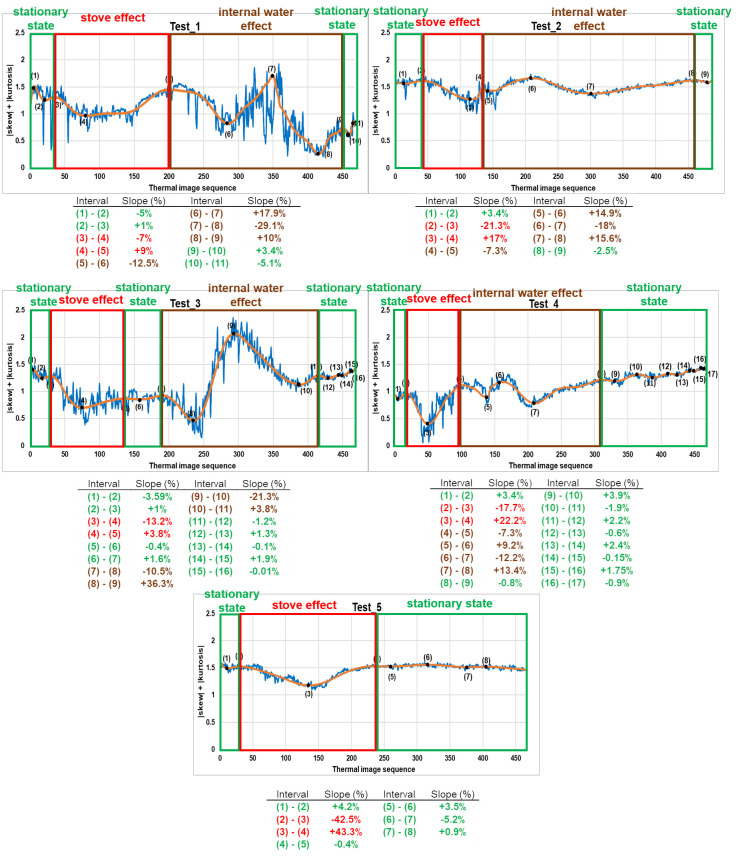
Relative maximums and minimums obtained (numbers indicated in the graph) and slope calculation (table below the graph) regarding the evolution of each test performed after SG smoothing filter application (orange line). Blue lines indicate the evolutions of the tests performed before SG smoothing filter application. The slopes belonging to the stationary state of the mosaic, the stove effect and the internal water effect (*w_shape*) are framed in green, red and brown rectangles in the graphs; and coloured with the same colours as their corresponding rectangles in the tables, respectively. The *reference_points* are the instants just after the beginning of internal water effect, i.e., the thermal images just after the extremes (5), (4), (7) and (4) of tests 1, 2, 3 and 4, respectively.

Analyzing the slopes in Figure 6, together with the information support of the steps followed in each test (Table 2), the following conclusions are reached, with the identification of phases in the evolution of the tests as illustrated in Figure 7:

1. The slopes not belonging to stove effect and internal water effect are similar to each other and close to 0% because during this time the mosaic under study is in a stationary state. The stove effect is understood as the period that includes the heating phase by the stove used in the tests of this work and the subsequent cooling phase, and internal water effect is understood as the time course from the first appearance of its thermal footprint until it no longer thermally alters the affected sample. In short, the evolution of the sum of skew and kurtosis after SG smoothing filter application is flat when the sample is thermally stable, with small increases and decreases due to the residual noise remained in the thermal images after the bilateral filtering application. Moreover, the values of skew and kurtosis in the quasi-zero slopes located before stove effect (and also after stove effect in Test_5) are generally close to zero but slightly higher. The reason for not being equal to zero, since at those moments of the evolution the material is in a stationary state and free of thermal footprints of defects, is due to the reflection and shading effects on the surface analysed. Such effects, as well as noise in thermal images, are very commonly found during the monitoring of cultural heritage.
2. The transmission mode partially mitigates the noise appearing in the thermal images due to the stove effect during its heating phase (transitory state). Possible reflection and shading effects are also mitigated by the measurement on transmission mode. By contrast, the cooling phase of the stove effect (transitory state) allows the sample under study to establish its original thermal behaviour. Therefore, there is an abrupt and prolonged slope for both the transitory heating (negative slope) and transitory cooling (positive slope). If the extremes of the slopes of stove effect of each test are compared with the instants of working of the stove, a certain time delay is appreciated due to the thermal mass of the sample (see Figure 7 and Table 2 for comparison purposes).
3. Moreover, there are two cycles of decrease—increase during the internal water effect, both cycles with prolonged and abrupt slopes. These four slopes represent an approximate *w-shape* (hereinafter, *w_shape*) explained by the following points:
  - The first descent of the *w_shape* is caused by an initial tendency to mitigate the effects of noise and possible reflections and shading from the first appearance of the thermal footprint of internal water (hereinafter, *reference_point*). The *reference_point* corresponds to the instant just after the starting point of this first descent. This mitigation is due to the fact that the thermal footprint of the internal water acts as a thermal stabilizer in its first stage of growth, with a practically negligible growth until the end of the first descent of the *w_shape*, while it tends to bring the absolute values of skew and kurtosis of the thermal images closer to zero.
  - Next, on the positive slope, the growth of the thermal footprint of the internal water begins to make its real effect on the absolute values of skew and kurtosis, which increase over time to the upper end of the slope. At the upper end of the first increase of the *w_shape*, the thermal footprint of the internal water reaches its maximum representation, where its growth is stabilized.
  - After that, the second and last descent of the *w_shape* tends again to bring the absolute skew and kurtosis values closer to zero, due to the inverse effect regarding the first descent of *w_shape*. In other words, the noise and the possible reflection and shading effects, which were mitigated along the first descent of *w_shape*, regain strength after the stabilization of the growth of the thermal footprint of the internal water, mitigating the effect of the internal water.
  - Finally, the noise and the possible reflection and shading effects continue to gain more strength in the thermal images corresponding to the last increase of the *w_shape*. Thus, the evolution of the sum of the absolute values of skew and kurtosis after SG smoothing filter application tends to have a behaviour similar to the instants prior the *reference_point* that do not correspond to the stove effect, but with a different average level due to the thermal effect of the presence of internal water.

If the *reference_point* of the *w_shape* of each test is compared with the real instant in which water is introduced into the mosaic, a certain time delay is appreciated (see Figure 7 and Table 2 for comparison purposes). This is due to the fact that the thermal diffusion always requires some time to reach the surface because of the thermal mass of the material. Furthermore, if the internal water effect and the stove effect coincide in time (Test_1, Test_2 and Test_4), the internal water effect superimposes on the stove effect.

As a result of the analysis performed in Step 1, Section 4.1 shows how the *reference_points* can be automatically detected in the case that the internal water appears by applying the same threshold value to the percentage values of the slopes in each of the tests, and by counting the number of consecutive slopes higher than that threshold value in each test. Note that the values of all slopes are converted to absolute terms when obtaining the results of Step 1.

### 3.2. Step 2: Automatic Delimitation of the Contour of the Thermal Footprint of Internal Water from Its First Appearance

In the affirmative case of detection of the *reference_point* after the result of the Step 1, and thus of the *w_shape* recognition, inequalities are searched in the shape of the histograms corresponding to the thermal images after the corresponding *reference_point*, including the latter, regarding the thermal image histogram corresponding to the instant just before the *reference_point*. It should be noted that the thermal images obtained after applying the bilateral filtering technique from Step 1 are also used in the analysis performed in the Step 2.

The search for these inequalities is performed by overlapping the thermal image histogram under study over the thermal image histogram corresponding to the instant just before the *reference_point*. In doing so, the detection of the pixel value belonging to the contour of the thermal footprint of the internal water of the image under study is possible through the automatic search for the point of intersection in the overlapping histograms (hereinafter, *intersection_point*). This *intersection_point* belongs to one of the ends of the Gaussian bell of the thermal footprint of the internal water according to the deduction made in Step 1, since the thermal image histogram just before the *reference_point* does not have a Gaussian bell of the thermal footprint of the internal water. In addition, with the objective of knowing on which side to search for the *intersection_point* in the overlapping histograms, the sign of the temperature difference between the volume of the internal water and of the unaltered surrounding is required. This way, the effect of noise, reflections and shading is eliminated. For this purpose, it is enough to measure the ambient temperature in the moment of study and know the standard value of temperature of the water sanitation network. Note that in most real cases the generation and propagation of moisture in the sample will be due to leaks from water pipes.

**Figure 8:**
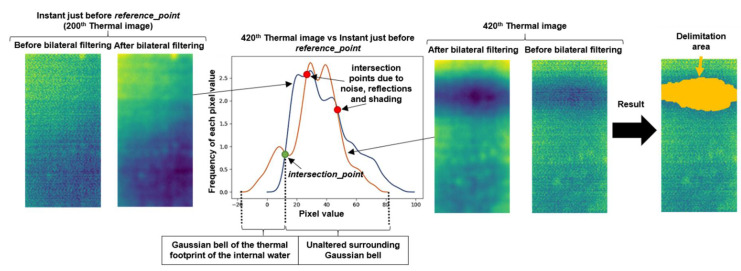
Step 2 result applied to the 420th thermal image belonging to the Test_1.

Figure 8 shows a result of this Step 2, taking an overlapping histograms in Test_1 as a reference. The results of all the tests are shown in Section 4.2.

Figure 8 shows the leftmost intersection point as the *intersection_point* in the overlapping histograms, since the temperature of the volume of internal water is colder than the temperature of the rest of the sample. The same happens to the rest of images of the Test_1, and also to the remaining sequences of this work (see ambient and water temperatures shown in Table 2). The knowledge of the *intersection_point* makes possible the delimitation of the contour of the thermal footprint of the internal water. In Figure 8, the area of the delimitation is highlighted in orange.

### 3.3. Step 3: Automatic Classification between Harmful and Non-Harmful Internal Water

The Step 3 of this study analyses again the slopes in the evolution of the sum of the absolute values of skew and kurtosis of each test performed after SG smoothing filter application, i.e., Figure 7. If the percentage values of the brown slopes are observed, i.e., the slopes corresponding to the internal water effect, it is possible to say that the values of the second and third slope of *w_shape* are the highest and the value of the last slope the lowest in the tests using *water_pipe*, in absolute terms. In contrast, the values of the third and fourth slope of *w_shape* are the highest and the value of the first slope the lowest in the tests using *water_sponge*, in absolute terms.

The main cause of this differentiation of the values of the slopes of the *w_shape*, in absolute terms, between the tests that generate internal water with *water_sponge* and with *water_pipe* is the different thermal diffusivity between the appearance of harmful and non-harmful internal water. Since non-harmful internal water is understood as the circulation of water through internal pipes, this circulation generates a forced flow of water increasing its thermal diffusivity to its environment several times, even though it is confined in a pipe that acts as a thermal barrier. The opposite happens with harmful internal water, since the propagation of moisture is not confined but not forced either, being quasi-stagnant and having a lower capacity of thermal diffusivity to its environment.

As a result of the analysis performed in Step 3, Section 4.3 shows how it is possible to automatically classify between harmful and non-harmful internal water by analysing which slopes of the *w_shape* are the highest and the lowest. Note that the values of all slopes are converted to absolute terms when obtaining the results of Step 3.

## 4. Results and Discussion

The results of each step of the data analysis of the tests performed in the mosaic under study are shown in the following subsections. Along with the results of each step, the corresponding discussion is conducted.

### 4.1. Step 1: Automatic Detection of the First Appearance of the Thermal Footprint of Internal Water

**Figure 9:**
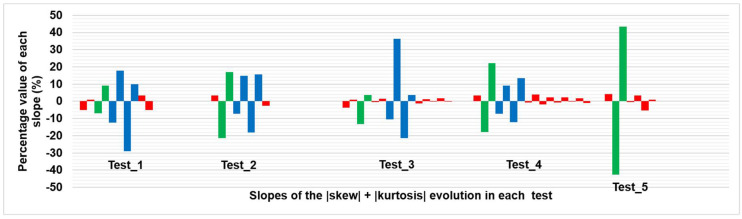
Alternative graphic representation of Figure 7.

Figure 9 shows another way to graphically represent Figure 7, in order to have a clearer and more direct visualization.

From Figure 9, it is easy to differentiate between the slopes corresponding to the stove effect and internal water effect and the slopes when the mosaic is in stationary state. Indeed, the corresponding average and standard deviation values are 2.1% ± 1.6% and 16.8% ± 10.7% considering all “red” and all “green + blue” bars of Figure 9, respectively. All the percentage values of the slopes are converted to absolute terms for the previous calculations and when setting a threshold value of 4.9% in order to automatically differentiate between “red” and “green + blue” bars. This 4.9% was determined as the intermediate value at the moment of the maximum approximation between the slopes of the stationary state (2.1% + 1.6% = 3.7%) and those of the stove effect and the internal water effect (16.8% − 10.7% = 6.1%).

Test_5 is used for the differentiation between the stove effect and the internal water effect, i.e., the “green” and “blue” bars of Figure 9, respectively, since in that test the internal water is not introduced in the sample. In the graph of Test_5, there are not four consecutive slopes with an absolute percentage value higher than 4.9%, i.e., there is no *w_shape*. Therefore, in the case of having four consecutive slopes with absolute percentage values higher than 4.9%, it can be stated that the thermal footprint of an internal water is affecting the surface of the material under study. Therefore, the *reference_point* is the instant immediately after the starting point of the first of the consecutive slopes. In the case of five or six consecutive slopes with an absolute percentage value higher than 4.9%, the last four belong to the *w_shape*, since the effect of the internal water is superimposed to the stove effect as explained at the end in Section 3.1.

It should be noted that in the case of having three consecutive slopes with absolute percentage slopes higher than 4.9%, these slopes are also associated with a *w_shape*, as it may be the case that the lowest slope of the *w_shape* has a value lower than the threshold value, being unlikely to have 3 different and consecutive transitory phases in a real situation. This is the case with Test_3, despite the previous calculations of the average and standard deviation. To automatically associate the fourth missing slope, the one with the highest value in absolute terms is associated as long as it has the right sign to correctly form the *w_shape*.

### 4.2. Step 2: Automatic Delimitation of the Contour of the Thermal Footprint of Internal Water from Its First Appearance

A quantitative analysis of the delimitations obtained in each test is performed with the purpose of showing the results according to the analysis in Step 2. The following performance metrics are used (Equations (5) to (7)):

*Precision* = *TP*/(*TP* + *FP*) (5)

*Recall* = *TP*/(*TP* + *FN*) (6)

*F*-*score* = 2 × (*Precision* × *Recall*)/(*Precision* + *Recall*) (7)

where *TP*, *FP* and *FN* are the True Positives, False Positives and False Negatives of the delimitation results, compared with the corresponding ground truths, respectively. The ground truths are obtained by an expert operator who has manually delimited each contour of the thermal footprint of the internal water in each thermal image sequence. It should be noted that the reason for using *F-score* is that this performance metric takes into account both the *precision* and *recall* parameters, thus being the most representative. In other words, *F-score* averages the performances of the detection (*precision*) and delimitation (*recall*) of the thermal footprint of the internal water in each result obtained.

**Figure 10:**
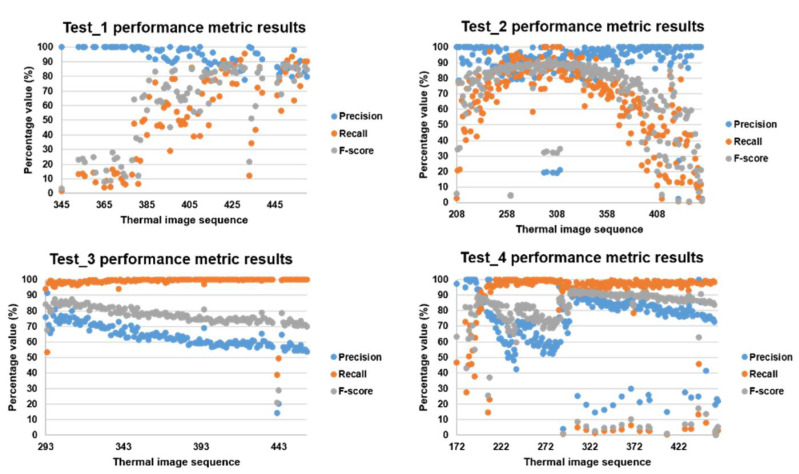
*Precision*, *Recall* and *F-score* results regarding the delimitations obtained in each test performed in this work.

**Figure 11:**
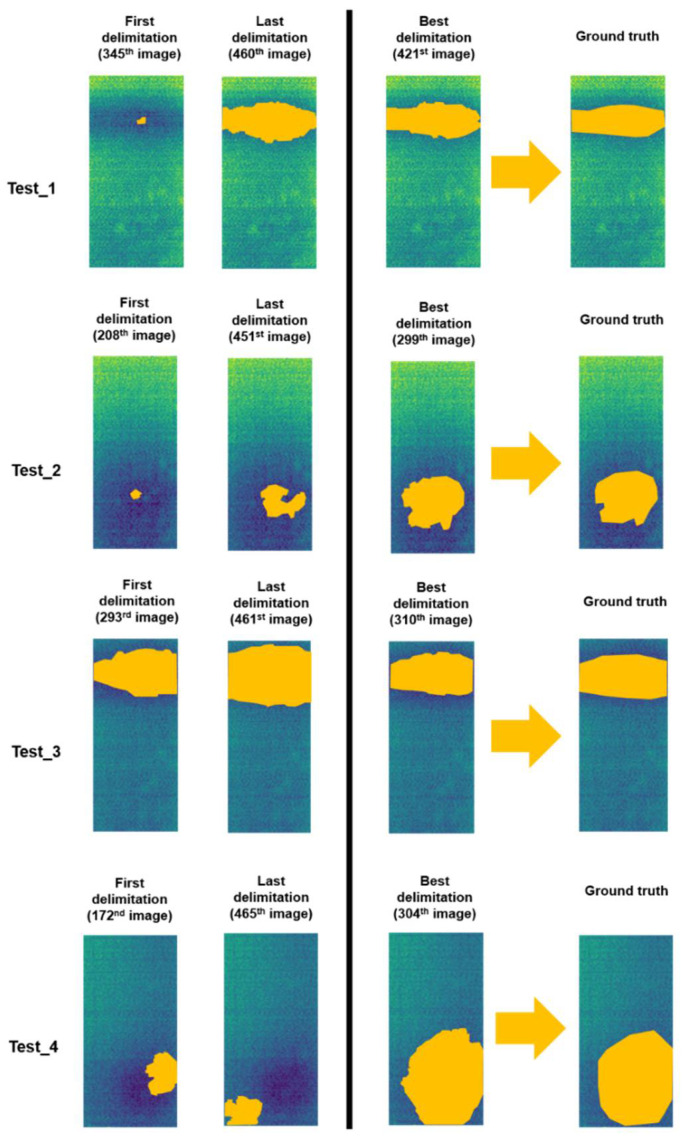
First and last delimitation obtained from each test. The best delimitation according to the *F-score* results together with the corresponding ground truth of each test are also indicated. All the delimitation areas are highlighted in orange.

Figure 10 shows different graphs, each corresponding to a different test, representing the corresponding *Precision*, *Recall* and *F-score* values obtained. In addition, with the aim at illustrating the values represented in the graphs of Figure 10, Figure 11 shows the first and the last delimitation obtained from each test, being the delimitation areas highlighted in orange on the corresponding raw thermal images. To illustrate how good the results obtained are according to the analysis in Step 2, Figure 11 also represents the best delimitation according to the *F-score* results together with the corresponding ground truth of each test (also highlighting the delimitation areas in orange).

**Figure 12:**
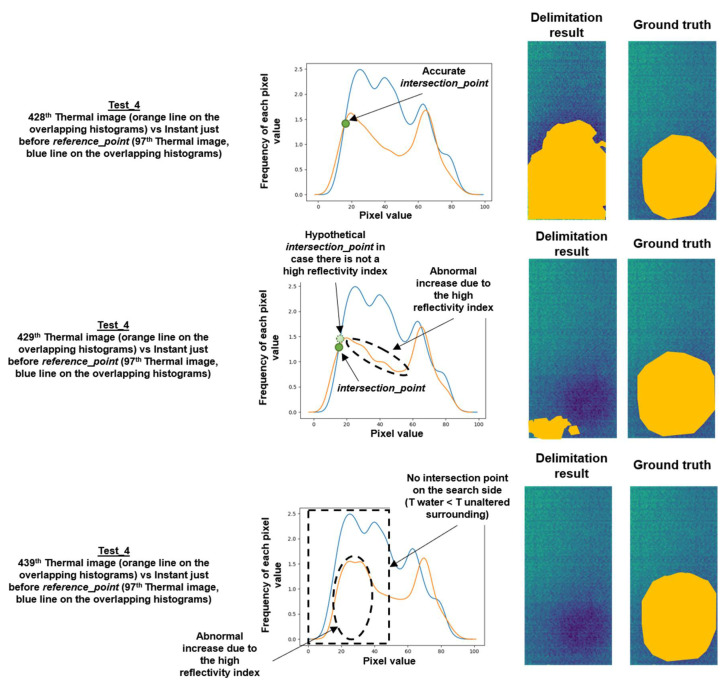
Examples of good and poor delimitation results. Delimitation areas are highlighted in orange.

It should be noted that the horizontal axis of each test in Figure 10 represents the results of the delimitation in the corresponding thermal images from the ending point of the first ascent of the *w_shape* within the sequence. The reason for showing from the ending point of the first ascent of the *w_shape*, and not from *reference_point* according to the analysis of the Step 2, is due to the very poor delimitation results obtained in the thermal images not represented in Figure 10. The cause of these poor results, along with not always having values equal to 100% in the performance metrics represented in Figure 10, is the high reflectivity index of the tested surface of the mosaic. Despite knowing on which side to search for the *intersection_point* in the overlapping histograms, this high reflectivity index may cause a small random variation in the shape of the region of the histograms located on the search side of the *intersection_point*. As a consequence, the *intersection_point* will be less accurate for the delimitation of the contour of the thermal footprint of the internal water, being the tested surface very sensitive to any thermal change in the sample environment. Moreover, the high reflectivity index may cause that there is no point of intersection on the search side on some overlapping histograms. So much that of the results represented in Figure 10, there are 29%, 1%, 5% and 11% of thermal images in Test_1, Test_2, Test_3 and Test_4 from the ending point of the first ascent of their corresponding *w_shapes* that have no delimitation and therefore are not represented in Figure 10, respectively. As an example, Figure 12 illustrates delimitations with a good and a poor result, highlighting the delimitation areas in orange, and a thermal image without delimitation.

Despite the problem of high reflectivity index, an issue that must be mitigated in the mosaic in future works, similar results are obtained in comparison with the results of methodologies that automatically delimit contours of the thermal footprints of moisture from single thermal images [18,22] both in *precision*, *recall* and *F-score*. Table 3 shows the average and standard deviation values of the performance metrics represented in Figure 10:

### 4.3. Step 3: Automatic Classification between Harmful and Non-Harmful Internal Water

Table 4 shows the percentage values of the slopes of *w_shape* in absolute terms, taking into account all tests with *reference_point* detected in Step 1.

As shown in Table 4, the two highest percentage values of the slopes of *w_shape* are marked in bold and the lowest one is underlined for each test. Then, according to the analysis in Step 3, the automatic classification between harmful and non-harmful internal water is possible by analysing which slopes are the highest and the lowest. Proof of this is the result shown in Table 5.

## 5. Conclusions

This work implies a step forward in the automation of the cultural heritage thermographic monitoring, allowing to optimize the decision-taking for rehabilitation actions in cultural heritage. For that purpose, a study for: (i) The automatic detection of the first appearance of the thermal footprint of internal water, together with; (ii) the automatic delimitation of the contour of the thermal footprint of internal water from its first appearance; and (iii) the automatic classification between harmful or non-harmful internal water, is proposed. To meet the above objectives, the study analyses a heritage element, having the corresponding thermal image sequences as input data.

Specifically, the heritage element under study is the mosaic described in [23,31], which has a *tessellatum* layer composed by synthetic tesserae, generating harmful or non-harmful internal water in four different tests and a fifth test without internal water generation for comparison purposes. Each test has focused on simulating a different real situation that can be found in the mosaic, acquiring a thermal image sequence for each test. Moreover, the study is based on the principles of the research line of [31], the only IRT paper that automates the cultural heritage thermographic monitoring so far.

Specifically, the nucleus of the study has focused on the automatic analysis of the temperature distribution of each thermal image in each test. The slopes of the evolution of the sum of the absolute values of skew and kurtosis of each thermal image of a sequence and the overlapping of the histograms of the thermal images from the instance just before the first appearance of the thermal footprint of internal water with respect to the histogram of that instant are analysed to fulfil the objectives; “(i) and (iii)” and “(ii)”, respectively.

This study offered positive analysis in the five tests performed. In the first four tests, the first appearance of the thermal footprint of internal water is automatically detected and the automatic classification between harmful and non-harmful internal water was successful, with the internal water being correctly undetected in the fifth test. With respect to delimitation, acceptable quantitative analysis has also been obtained when compared with other automation IRT methodologies that work with single thermal images. Even so, the high reflectivity index of the tested surface of the mosaic negatively affects some overlapping histograms, affecting to all the sequences performed.

Future researches will deal with the application of this study to other mosaics and other types of heritage elements under real conditions, in order to refine a standard procedure for real cases. In addition, the detection of other types of defects will be studied, in order to make the study more robust for cases where several defects are present. Moreover, future works will also try to combat the reflectivity index when monitoring a surface as reflective as the *tessellatum* layer of the mosaic tested in this study, which prejudices the performance of the automatic delimitation.

## Figures and Tables

**Table 1:** Specifications of the thermographic camera used.

Sensor Type	Uncooled Focal Plane Array (μbolometer)
Thermal image/pixels	640 (H) × 480 (V)
Resolution (°C)	0.1
Accuracy	±2 °C or ±2% of reading, whichever is greater
Spectral ranges (μm)	8 @ 14

**Table 2:** Steps followed in the experiments performed.

Tests	Time in s (Minutes between Brackets)					
	0 (0)	180 (3)	600 (10)	780 (13)	840 (14)	2340 (39)
Test_1	Camera ON Ta ^1^ = 22.7 RH ^2^ = 45.5	Stove ON	-	Stove OFF	Water pump ON (T_H2O_^3^ = 11)	Camera OFF Water pump OFF (T_H2O_ = 17.7)
Test_2	Camera ON Ta = 25.2 RH = 43.3				Water injection to sponge (50 mL, T_H2O_ = 15)	Camera OFF
Test_3	Camera ON Ta = 24.8 RH = 39.4		Water pump ON (T_H2O_ = 10.8)		-	Camera OFF Water pump OFF (T_H2O_ = 20.5)
Test_4	Camera ON Ta = 24.9 RH = 40.4		Water injection to sponge (50 mL, T_H2O_ = 11)			Camera OFF
Test_5	Camera ON Ta = 26.5 RH = 48.8		-			Camera OFF

^1^ Ambient temperature (°C). ^2^ Relative humidity (%). ^3^ Water temperature (°C).

**Table 3:** Mean and standard deviation values of the performance metrics regarding the Figure 10.

Performance Metrics	Test_1	Test_2	Test_3	Test_4
*Precision* (%)	93 ± 6	91 ± 17	64 ± 9	74 ± 19
*Recall* (%)	54 ± 29	65 ± 25	98 ± 7	87 ± 27
*F-score* (%)	62 ± 26	71 ± 23	77 ± 7	78 ± 22

**Table 4:** Percentage values of the slopes of *w_shape* in absolute terms. The two highest values are marked in bold and the lowest value is underlined in each test.

Tests	Percentage Values of the Slopes of *w_shape* in Absolute Terms (%)			
	1st Slope	2nd Slope	3rd Slope	4th Slope
Test_1	12.5	**17.9**	**29.1**	10
Test_2	7.3	14.9	**18**	**15.6**
Test_3	10.5	**36.3**	**21.3**	3.8
Test_4	7.3	9.2	**12.2**	**13.4**

**Table 5:** Slopes of *w_shape* classified from highest to lowest (from left to right).

Tests	Slopes of *w_shape* Classified from Highest to Lowest		
Test_1 and Test_3 (not harmful)	2nd and 3rd or 3rd and 2nd	1st	4th
Test_2 and Test_4 (harmful)	3rd and 4th or 4th and 3rd	2nd	1st
